# The Relationship Between School Climate and Mental and Emotional Wellbeing Over the Transition from Primary to Secondary School

**DOI:** 10.1186/s13612-015-0037-8

**Published:** 2015-10-22

**Authors:** Leanne Lester, Donna Cross

**Affiliations:** Health Promotion Evaluation Unit, The University of Western Australia, Perth, WA Australia; Telethon Kids Institute, The University of Western Australia, Perth, WA Australia

## Abstract

**Background:**

School climate has often been described as the “quality and character of school life”, including both social and physical aspects of the school, that can positively promote behaviour, school achievement, and the social and emotional development of students.

**Methods:**

The current study examined the relationship between students’ mental and emotional wellbeing and factors pertaining to school climate, focussing on the domains of safety, social relationships and school connectedness, during the last year of their primary schooling (age 11–12 years) and their first 2 years of secondary school. Data was collected using a self-completion questionnaire, four times over 3 years from 1800 students’ aged 11–14 years. Multilevel modelling was used to determine the strongest school climate predictor of students’ mental and emotional wellbeing at each time point.

**Results:**

In the last year of primary school, peer support was the strongest *protective* predictor of wellbeing, while feeling less connected and less safe at school predicted mental wellbeing. Feeling safe at school was the strongest *protective* factor for student wellbeing in the first year of secondary school. In the second year of secondary school, peer support was the strongest *protective* factor for mental wellbeing, while feeling safe at school, feeling connected to school and having support from peers were predictive of emotional wellbeing.

**Conclusions:**

School climate factors of feeling safe at school, feeling connected to school, and peer support are all protective of mental and emotional wellbeing over the transition period while connectedness to teachers is protective of emotional wellbeing. Primary school appears to be an important time to establish quality connections to peers who have a powerful role in providing support for one another before the transition to secondary school. However, school policies and practices promoting safety and encouraging and enabling connectedness are important during the first years of secondary school.

Recommendations for effective school policy and practice in both primary and secondary schools to help enhance the mental and emotional wellbeing of adolescents are discussed.

## Background

As the concepts of school climate and school culture are related, they are often used interchangeably. School climate has been defined by Cohen and colleagues (2009a, b) as the character and quality of life within a school and refers not only to the physical environment but also to the whole school experience, whereas school culture refers to a set of beliefs or values. School climate has been described as the leverage for school culture (Gruenert, 2008). Five common school climate domains have previously been identified: order, safety, and discipline; academic outcomes; social relationships; school facilities; and school connectedness (Zullig, Koopman, Patton, & Ubbes, 2010). More recently, the school improvement process has also been identified as an important dimension of school climate (Thapa, Cohen, Guffey, & Higgins-D’Alessandro, 2013).

A sustained positive school climate promotes student social, mental and emotional development, and behavioural and learning outcomes, while guaranteeing both physical and social safety (Loukas & Robinson, 2004; Zullig et al., 2010). Research has shown positive school climate is associated with improved academic achievement and performance, adaptive psychosocial adjustment, satisfaction with school, sense of belonging, academic value and self-concept, motivation to learn, decreased behavioural problems and overall positive health and wellbeing (MacNeil, Prater, & Busch, 2009; Roeser, Eccles, & Freedman-Doan, 1999; Vieno, Perkins, Smith, & Santinello, 2005; Wang, Selman, Dishion, & Stormshak, 2010; Zullig, Huebner, & Patton, 2011). Positive school climate can also reduce teacher burnout, promote teacher retention, and can also enhance parent-school partnerships (Cohen, McCabe, Michelli, & Pickeral, 2009; Grayson & Alvarez, 2008).

This current study investigated students’ perception of components of school climate that can effect their mental and emotional wellbeing outcomes as they transition from primary to secondary school. This transition to secondary school is a period of life known to affect the psychological, social and intellectual wellbeing of students and is aptly described as ‘one of the defining parameters of development in the second decade of life’ (Barber & Olsen, 2004b). It is also considered to be one of the most challenging times to match developmental needs with school structures (Brinthaupt, Lipka, & Wallace, 2007). In this period of rapid physical, social and emotional development for adolescents, the change in school and social structures can result in increased feelings of loneliness and isolation, victimisation, and negative and disruptive behaviours (Cohen & Smerdon, 2009; Cross et al., 2009). Students move from a relatively structured primary school setting often characterised by smaller class sizes, where they are often the oldest students in a school, in a smaller student cohort, and a classroom structure where students are taught by one main classroom teacher, into a larger secondary school where they are the youngest students and move between classes and teachers across the school day. Australian secondary schools usually have larger student cohorts (range 30–180) and employ specialist teaching staff who teach 25–30 students for between 30 and 80 min before they move to their next class. At some point during the school day, students usually meet with the same group of 20 peers and one teacher who is the primary provider of students’ pastoral care. These differences in primary and secondary school structures are not unique to Australian schools.

Social relationships dominate the school transition experience (Pereira & Pooley, 2007) with students often needing to develop new friendships and define their place in a new social hierarchy (Pellegrini & Bartini, 2000) while having an increased reliance on their peer group for social support. Social factors which have been identified as protective over the transition period include the ability to make new friends (Akos & Galassi, 2004), the number and quality of friends (Pellegrini & Bartini, 2000), peer support (Pellegrini, 2002), liking school (Barber & Olsen, 2004a), school belonging (Benner & Graham, 2009), connectedness to school (O’Brennan & Furlong, 2010) and feeling safe at school (Espelage, Bosworth, & Simon, 2000).

The transition period can also be an especially vulnerable time for adolescents as it also coincides with the onset of many depressive and anxiety disorders (Hankin & Abramson, 2001). As outlined above, the school climate components of relationships, and sense of safety, and belonging to the school are important interrelated factors during transition. These components are examined to determine if they are predictors of students’ mental and emotional wellbeing in this study”.

Close relationships to teachers and peers in primary school predicts a positive transition into secondary school (Waters, Lester, & Cross, 2014a) and are associated with students reporting fewer emotional problems, feelings of depression and anxiety and use of anti-social behaviours (Frey, Ruchkin, Martin, & Schwab-Stone, 2009; Kidger, Araya, Donovan, & Gunnell, 2012). Having and valuing peer support also enhances feelings of school safety (Cowie & Oztug, 2008) with students’ perception of safety at school negatively influenced by bullying. Victimisation at primary school is associated with lower feelings of safety and school connectedness at secondary school following the transition from primary school (Bradshaw, O’Brennan, & Sawyer, 2008). Poor teacher and peer relationships, a lack of peer support, bullying, victimisation, and higher safety concerns are related to declines in psychological adjustment such as self-esteem, and mental health problems such as symptoms of depression, anxiety, and suicidality (Kuperminc, Leadbeater, & Blatt, 2001; Loukas & Robinson, 2004; Ozer & Weinstein, 2004; Smith & Brain, 2000; Way, Reddy, & Rhodes, 2007).

School connectedness or a sense of belonging describes the quality of the social relationships within the school: the extent to which a student feels like he/she belongs at school and feels cared for by the school (McNeely, Nonnemaker, & Blum, 2002), and is associated with higher academic achievement, good attendance, social relationships, and increased mental and emotional wellbeing of students (Bond et al., 2007; Kuperminc et al., 2001; McNeely et al., 2002). Higher levels of school connectedness in students is influenced by a smoother secondary school transition, and fewer classroom, peer and emotional problems (Waters, Cross, & Shaw, 2010) whereas social isolation, feeling unsafe at school, and poor classroom management are a threat to school connection (Blum, 2005).

To address the lack of empirical research to understand students’ perceptions of school climate and related outcomes, a recent study by Hung and colleagues (2014) focussed on student perceptions of school climate as predictors of victimisation, and emotional and conduct problems over the transition from primary to secondary school. This study found the school climate factors ‘Authoritative Structure’ and ‘Student Order’ were each uniquely and inversely related to emotional and conduct problems as well as victimisation (Hung et al., 2014).This study aims to add to the empirical evidence by exploring the hypothesis that students’ who are transitioning from primary to secondary school who have positive perceptions of the school climate, especially the quality of their social relationships, their connectedness to their school and their safety will have higher levels of mental and emotional wellbeing. This study will also examines how each school climate factor differentially predicts students’ mental and emotional wellbeing.

## Methods

The data in this study were collected as part of a larger group randomised control (longitudinal) study, called the Supportive Schools Project (SSP) conducted in Perth, Western Australia. This study aimed to develop and implement whole-of-school strategies to reduce the prevalence of frequent bullying behaviours, as well as positively influence common mediators of bullying. Data from only the study comparison schools are used in this paper, as the SSP intervention is not a focus of this paper. The study was approved by the Edith Cowan University Human Research Ethics Committee and the relevant Catholic Education school ethics authorities.

### Sampling and Data Collection

To reduce the rate of transition attrition as students move from primary to secondary schools, secondary schools affiliated with the Catholic Education Office (CEO) of Western Australia were recruited to participate in the study. Students attending Catholic schools in Australia are more likely than students attending schools in other sectors (e.g., government schools) to move from primary to secondary schools in intact groups.

Cohort data were collected during the Supportive Schools Project (SSP) from 3462 students from 21 of the 28 Catholic secondary schools in Western Australia. The seven schools that declined to participate in the study cited other priorities within their school and demanding staff workloads. All invited CEO schools were stratified according to the total number of students enrolled at the school and each school’s Socio-Economic Status (SES) top help to control for the influence of these two factors on student bullying behaviour. These schools were randomly selected and randomly assigned to an intervention or comparison group (Cross, Hall, Waters, & Hamilton, 2008). The data used in this paper was collected from 1800 students assigned to comparison schools (n = 11 schools) in four waves from 2005 to 2007. To collect data relating to their pre-transition experience, all primary students enrolled to commence secondary school at each of the 21 participating secondary schools received a baseline survey while in their respective primary schools. Parents of secondary students at the 21 secondary schools, who had not been recruited in primary school (as they were not on the school enrolment lists) were approached for consent for their child’s participation at the first follow-up.

Active consent (where parents gave written permission for their child to participate) was requested from all parents, if any parents did not respond to this active consent approach up to two follow-up letters were mailed to parents requesting their passive consent where they were required to opt-out if they did not wish their child to participate (Ellickson & Hawes, 1989). This two layered consent process resulted in 93 % of parents whose children were enrolled in the 21 recruited secondary schools consenting to their child participating in the study.

The student cohort was surveyed at the end of primary school (mean age 12 years), the beginning and end of the first year of secondary school (mean age 13 years old) and the end of the second year of secondary school (mean age 14 years old). In total, 1810 comparison students completed questionnaires at least at one time point with 1650 (91 %) responding to at least three of the four data collection points. One half of the students surveyed were male and 70 % attended a co-educational secondary school versus a single sex secondary school. Responses from only the students from the SSP study comparison schools at the end of primary school, first year secondary school and second year of secondary school were used in the analysis detailed below.

### Measures

School climate was represented using four measures: safety at school, connectedness to teachers, connectedness to school, and peer support.

#### Safety

Safety at school was a single item adapted from the Peer Relations Questionnaire (Rigby & Slee, 1998) and measured on a three point scale (1 = no, I never feel safe at school, 2 = yes, some of the time, 3 = yes, all or most of the time) for each time point with a higher value reflecting greater feelings of safety at school.

#### Teacher Connectedness

The teacher connectedness to school was from the Resnick, Bearman, Blum, Bauman, Harris, Jones, Tabor, Beuhring, Sieving, Shew, Ireland, Bearinger, & Udry (1997) six item Teacher Connectedness Scale (At my school, there is a teacher or some other adult who: Really cares about me; Tells me when I do a good job; Would notice when I’m not there; Always wants me to do my best; Listens to me when I have something to say; Believes that I will be successful) measured on a five point scale (1 = unsure, 2 = never, 3 = some of the time, 4 = Most of the time, 5 = all of the time). The unidimensionality of the adapted scale was confirmed in a factor analysis (CFI > 0.9, SMR < 0.10). For each student an average teacher connectedness score was calculated, with a higher score reflecting greater feelings of connectedness to their teacher (average alpha = 0.81).

#### Connectedness to School

The connectedness to school scale comprised four items adapted from the Resnick et al. (1997) six item School Connectedness Scale (I feel close to people at school; I feel like I am part of this school; I am happy to be at school; the teachers treat students fairly) measured on a five point scale (1 = never, 2 = unsure, 3 = sometimes, 4 = usually, 5 = always). The unidimensionality of the adapted scale was confirmed in a factor analysis (CFI > 0.9, SMR < 0.10 at all time points). For each student at each time point an average school connectedness score was calculated, with a higher score reflecting greater feelings of connectedness to their school (average alpha = 0.80).

#### Peer Support

The peer support at school scale (adapted from the 24-item Perceptions of Peer Social Support Scale; (Ladd, Kochenderfer, & Coleman, 1996) comprised eleven items: How often would students: choose you on their team; tell you you’re good at things; explain something if you didn’t understand; invite you to do things with them; help you if you are hurt; miss you if you weren’t at school; help you if something is bothering you; ask to work with you; help you if other students treat you badly; ask you to join in when alone; and share things with you? Items were measured on a three point scale (1 = never, 2 = sometimes, 3 = lots of times). A factor analysis performed on the adapted peer support scale confirmed its unidimensionality (CFI > 0.9, SMR < 0.10 at all time points). A peer support score at each time point was calculated for each student by averaging all items, higher scores reflecting greater feelings of peer support (average alpha = 0.88).

#### Emotional Wellbeing

The Strengths and Difficulties Questionnaire (SDQ) is a 25-item behavioural screening tool appropriate for use with 4 to 17 year olds (Goodman, 1997) and uses a three point scale (“0 = not true, 1 = somewhat true, 2 = certainly true”). The SDQ measures strengths (10 items) and difficulties (15 items) over the last month and comprises five subscales: emotional symptoms (average alpha = 0.70); conduct problems (average alpha = 0.40); hyperactivity (average alpha = 0.62); peer problems (average alpha = 0.46); and pro-social behaviour (average alpha = 0.70). The subscales and an overall score were calculated in accordance with the scale author’s instructions.

#### Mental Wellbeing

The Depression Anxiety Stress Scales-21 (DASS-21) is a 21-item self-report inventory composed of three subscales comprising seven items related to depression, anxiety and stress. The DASS-21 uses a four-point Likert scale from 0 (“did not apply to me at all”) to 3 (“applied to me very much or most of the time”) to indicate the extent to which an individual has experienced each affective state during the past week (Lovibond & Lovibond, 1995). A depression (average alpha = 0.90), anxiety (average alpha = 0.85), and stress (average alpha = 0.87) score was calculated at each time point for each student by adding the items. Higher scores reflect greater levels of distress.

### Statistical Analysis

SPSS v 22 and Stata v 13 were used to analyse the data longitudinally. Repeated measures models were used to determine differences over time for individual level perceptions of school climate and mental and emotional wellbeing. Separate regression models were used to determine the school climate (feeling safe at school, feeling connected to school, feeling connected to teachers, peer support) predictors of mental and emotional wellbeing at the end of primary school, first year of secondary school and second year of secondary school, while accounting for gender. First year secondary school models took into account primary school climate measures, whereas second year of secondary school models took into account first year of secondary school school climate measures. A random intercept was included in each regression model to account for the clustering of students within schools.

## Results

On average students felt safe at school, felt connected to school and their teachers, and felt supported by their peers (Table 1). Whereas a significant decline in students’ perception of safety at school, and feeling connected to school and teachers occurred after the transition into secondary school, levels of peer support remained constant over the three time points. Depression, anxiety, emotional problems, conduct problems and total difficulties significantly increased after the transition into secondary school, whereas peer problems and pro-social tendencies significantly decreased after the transition into secondary school.

**Table 1:** Descriptive statistics of school climate measures and mental and emotional wellbeing factors

Mean (sd)	End of Primary school (n = 1054)	End of first year secondary school (n = 1718)	End of second year secondary school (n = 1616)
School climate measures			
Safety at school (1–3)**	2.83 (0.39)	2.70 (0.51)	2.71 (0.51)
School connectedness (1–5)**	4.42 (0.58)	4.14 (0.76)	4.00 (0.84)
Teacher connectedness (1–5)**	4.10 (0.81)	3.79 (1.03)	3.63 (1.14)
Peer support (1–3)	2.58 (0.33)	2.56 (0.40)	2.58 (0.42)
Mental wellbeing (DASS)			
Depression (1–7)**	3.58 (6.03)	5.15 (8.60)	5.91 (9.39)
Anxiety (1–7)**	3.13 (4.96)	4.19 (7.36)	4.55 (7.85)
Stress (1–7)**	5.99 (6.92)	5.80 (8.24)	6.57 (8.79)
Emotional wellbeing (SDQ)			
Emotional problems (1–5)**	2.16 (2.11)	2.41 (2.44)	2.55 (2.46)
Conduct problems (1–5)**	1.64 (0.73)	1.93 (1.92)	2.09 (2.05)
Hyperactivity (1–5)	3.50 (1.22)	3.47 (2.44)	3.78 (2.45)
Peer problems (1–5)**	2.48 (0.79)	1.47 (1.67)	1.51 (1.75)
Prosocial (0–10)**	8.42 (1.57)	7.73 (1.98)	7.52 (2.16)
Total SDQ (0–40)**	8.03 (2.50)	9.28 (6.50)	9.92 (6.74)

** p < 0.001 for end of first and second year of secondary school compared to end of primary school

Safety at school, school connectedness and peer support were all significant predictors of mental wellbeing at the end of primary school (Table 2). School connectedness was the most significant protective factor against depression (β = −2.28), while peer support was the most significant predictor against anxiety (β = −1.56) and stress (β = −2.97). Peer support and school connectedness were protective against all emotional difficulties subscales, and peer support was the most significant protective factor of these two component of school climate. Feeling safe at school was protective against all emotional difficulties subscales, while feeling connected to teachers was protective against conduct problems, hyperactivity and peer problems, and was predictive of pro-social behaviour.

**Table 2:** School climate predictors of mental and emotional wellbeing at the end of primary school, first year secondary school and second year secondary school

	Safety at school		School connectedness		Teacher connectedness		Peer support	
	β	95 % Confidence interval	β	95 % Confidence interval	β	95 % Confidence interval	β	95 % Confidence interval
End of primary school								
Mental wellbeing (DASS)								
Depression	−1.80	(−2.73, −0.86)**	−2.28	(−3.08, −1.48)**	−0.11	(−0.61, 0.39)	−2.13	(−3.41, −0.84)**
Anxiety	−1.48	(−2.27, −0.68)**	−1.22	(−1.84, −0.54)**	−0.01	(−0.44, 0.41)	−1.56	(−2.64, −0.47)**
Stress	−2.36	(−3.44, −1.27)**	−1.78	(−2.71, −0.86)**	−0.05	(−0.64, 0.53)	−2.97	(−4.46, −1.48)**
Emotional wellbeing (SDQ)								
Emotional symptoms	−0.79	(−1.11, −0.47)**	−0.67	(−0.94, −0.39)**	0.14	(−0.03, 0.32)	−1.25	(−1.69, −0.81)**
Conduct problems	−0.15	(−0.26, 0.03)*	−0.16	(−0.26, −0.07)**	−0.07	(−0.13, −0.01)*	−0.19	(−0.34, −0.03)*
Hyperactivity	−0.34	(−0.53, −0.15)**	−0.22	(−0.38, −0.05)**	−0.20	(−0.30, −0.10)**	−0.36	(−0.62, −0.10)**
Peer problems	−0.24	(−0.35, −0.13)**	−0.34	(−0.43, −0.24)**	0.07	(0.01, 0.13)*	−0.82	(−0.97, −0.67)**
Pro-social behaviour	0.19	(−0.04, 0.43)	0.39	(0.19, 0.59)**	0.36	(0.24, 0.49)**	0.72	(0.39, 1.04)**
Total difficulties	−0.99	(−1.34, −0.63)**	−0.98	(−1.29, −0.68)**	−0.12	(−0.31, 0.07)	−1.80	(−2.30, −1.31)**
End of first year secondary school								
Mental Health (DASS)								
Depression	−3.03	(−4.02, −2.04)**	−3.14	(−3.91, −2.38)**	−0.22	(−0.70, 0.25)	−0.50	(−1.80, 0.80)
Anxiety	−4.01	(−4.84, −3.18)**	−1.39	(−2.03, −0.75)**	0.01	(−0.38, 0.40)	−0.66	(−1.73, 0.43)
Stress	−2.97	(−3.94, −1.99)**	−2.58	(−3.32, −1.82)**	0.03	(−0.44, 0.49)	−0.51	(−1.78, 0.77)
Emotional wellbeing (SDQ)								
Emotional symptoms	−0.74	(−1.04, −0.45)**	−0.63	(−0.85, −0.40)**	−0.05	(−0.19, 0.08)	−0.65	(−1.04, −0.26)**
Conduct problems	−0.61	(−0.85, −0.37)**	−0.36	(−0.55, −0.18)**	−0.25	(−0.36, −0.14)**	−0.04	(−0.36, 0.28)
Hyperactivity	−0.19	(−0.50, 0.13)	−0.70	(−0.94, −0.46)**	−0.32	(−0.47, −0.17)**	−0.26	(−0.68, 0.15)
Peer problems	−0.57	(−0.77, −0.37)**	−0.45	(−0.61, −0.30)**	−0.01	(−0.11, 0.08)	−1.39	(−1.66, 1.12)**
Pro-social behaviour	−0.12	(−0.35, 0.12)	0.27	(0.09, 0.45)**	0.32	(0.20, 0.43)**	0.93	(0.61, 1.24)**
Total difficulties	−2.05	(−2.78, −1.31)**	−2.11	(−2.67, −1.54)**	−0.62	(−0.97, −0.28)**	−2.05	(−3.02, −1.07)**
End of second year secondary school								
Mental wellbeing (DASS)								
Depression	−2.48	(−3.40, −1.56)**	−2.70	(−3.34, −2.06)**	0.37	(−0.03, 0.77)	−2.85	(−4.02, −1.68)**
Anxiety	−2.02	(−2.81, −1.24)**	−1.82	(−2.37, −1.28)**	0.21	(−0. 12, 0.55)	−2.90	(−3.89, −1.92)**
Stress	−2.13	(−3.00, −1.26)**	−2.12	(−2.72, −1.52)**	0.42	(0.04, 0.79)*	0.37	(0.32, 0.42)**
Emotional wellbeing (SDQ)								
Emotional symptoms	−0.72	(−0.96, −0.48)**	−0.55	(−0.71, −0.39)**	0.02	(−0.08, 0.12)	−0.38	(−0.68, −0.08)*
Conduct problems	−0.35	(−0.55, −0.15)*	−0.54	(−0.67, −0.40)**	−0.07	(−0.16, 0.38)	−0.13	(−0.12, −0.38)
Hyperactivity	−0.44	(−0.67, −0.21)**	−0.45	(−0.61, −0.30)**	−0.19	(−0.28, −0.09)**	−0.21	(−0.08, 0.50)
Peer problems	−0.51	(−0.67, −0.34)**	−0.34	(−0.45, −0.23)**	−0.05	(−0.12, 0.02)	−1.09	(−1.30, −0.88)**
Pro-social behaviour	0.13	(−0.08, 0.34)	0.39	(−0.24, 0.57)**	0.20	(0.11, 0.29)**	0.64	(0.38, 0.91)**
Total difficulties	−1.94	(−2.52, −1.36)**	−1.82	(−2.22, −1.41)**	−0.32	(−0.57, −0.07)*	−0.90	(−1.63, −0.15)*

Models at the end of primary school controlled for gender. All models at the end of first and second year of secondary school controlled for gender and measures at the previous time point

* Significant at p < 0.05

** Significant at p < 0.01

At the end of the first year of secondary school, safety at school and connectedness were significant predictors of mental wellbeing (Table 2). Somewhat similar to the primary school results, school connectedness was the most significant protective factor against depression (β = −3.14), whereas feeling safe at school was the most significant protective factor against anxiety (β = −4.01) and stress (β = −2.97). School connectedness was protective for all emotional difficulties subscales. However, feeling safe at school was most protective against emotional symptoms (β = −0.74) and conduct problems (β = −0.61).

At the end of second year of secondary school, feeling safe at school, feeling connected to school, and peer support were significant predictors of mental wellbeing. Peer support was the most significant protective factor against depression (β = −2.28) and anxiety (β = −2.90), while feeling safe at school (β = −2.13) and feeling connected to school (β = −2.12) was the most significant protective factor against stress. Feeling safe at school and connected to school were significant protective factors for all emotional difficulties scales. Connectedness to teachers was protective against hyperactivity and total difficulties and predictive of pro-social behaviour. Peer support was protective against peer problems and predictive of pro-social behaviour.

## Discussion

Student perceptions of school climate have been found to contribute to positive academic, social and emotional outcomes (Blum, Libbey, Bishop, & Bishop, 2004), but as highlighted by Hung et al., (2014), limited research has examined middle school student perceptions of school climate factors on student mental health and social wellbeing outcomes. This research examined interrelated individual-level school climate factors which have been found to be significant during the transition from primary to secondary school and determine their differential impact on students’ mental and emotional wellbeing. While there is limited consensus around a single definition of wellbeing, there is general agreement that at a minimum, social and emotional wellbeing includes the presence of positive emotions and moods and the absence of negative emotions and mental health disorders (AIHW, 2012).

As was expected, individual-level school climate measures of feeling safe at school, feeling connected to school and teachers, and peer support dropped after transitioning into secondary school. While transitioning into secondary school is a positive experience for most students (Waters, Lester, & Cross, 2014b), for some students the change in school context is marked by social, academic and structural concerns and can be the beginning of school disconnection and academic disengagement (Cohen & Smerdon, 2009). This study found that while feeling safe at school, feeling connected at school and peer support were significant predictors of mental and emotional wellbeing, peer support was the most significant protective factor over the transition period from primary to secondary school. Peer relationships at school have been found to contribute most to students’ wellbeing (Weare & Gray, 2003) as social and emotional challenges during the transition period can translate to frustration and anxiety causing negative or disruptive behaviours (Cohen & Smerdon, 2009). Social challenges during the transition period can include increased feelings of isolation as friendship groups change and adolescents develop new friendships and lose friends at a time when great importance is placed on peer relationships (Pellegrini & Bartini, 2000). Support from your peers was also the most significant predictor of positive transition expectations, and is a contributor to the actual transition experience (Waters et al., 2014b).

At the end of the first year of secondary school, feeling safe at school and feeling connected to school were the individual-level school climate factors most protective of mental and emotional wellbeing. Feeling safe at school includes social-emotional safety, physical safety, and substance use (Bradshaw, Waasdorp, Debnam, & Johnson, 2014) and is associated with academic, behavioural, social, emotional, and physical wellbeing (Reiss & Roth, 1993). To enhance students’ feelings of safety the school’s sociological and organisational structures can be modified by having fairer and more consistently applied and transparent school discipline policies and by increasing teacher and adult support for students (Samdal, Nutbeam, Wold, & Kannas, 1998).

School connectedness describes the quality of the social relationships within the school, and the extent to which students feel like they belong and feel cared for by people at their school (McNeely et al., 2002). Interventions to improve students’ school connectedness at the beginning of secondary school need to focus on the quality of the school’s pastoral care strategies and physical environment (Waters et al., 2010). Pastoral care strategies include the promotion of health and wellbeing, resilience, academic care, and social capital (Nadge, 2005; Quigley, 2004; WHO, 1998) through the implementation of school policies and programs at the school, teacher, student and school-community levels (Hearn, Campbell-Pope, House, & Cross, 2006). Students school connectedness can also be increased by encouraging them to achieve their highest academic potential and to participate in extracurricular activities such as sport, recreation, music, arts and service (Hamilton, Cross, Hall, & Townsend, 2003; Waters et al., 2010). The school’s built environment and the care taken by the school community to maintain the school grounds can also have an impact on students’ connectedness with the school (Waters et al., 2010).

The pattern of individual-level school climate effects were similar between end of primary school and second year of secondary school. In the first year of secondary school, students did not receive as much protective benefit from peers. The benefit received from having peers in primary school seems to have been lost after the move to secondary school and may have taken up to 2 years to redevelop highlighting the importance of peer support activities both pre-transition and during first year secondary school. Transition coincides with the adolescent developmental shift from a reliance on parents to a reliance on peers (Pereira & Pooley, 2007) with peer support needed for the development of social, emotional and mental health (McGraw, Moore, Fuller, & Bates, 2007). However, an increase in bullying behaviour also appears to occur in the immediate transition period from primary school to secondary school as students define their place in a new social hierarchy (Cross et al., 2009). Successful whole school interventions to increase peer support and decrease bullying during the transition period have included: encouraging student interaction between families, teachers and students; student counselling services; encouraging effective social interaction and social competence; and designing curriculum content to encourage co-operative and helpful behaviour and support of peers (Buchanan & Bowen, 2008; Denham, Wyatt, Bassett, Echeverria, & Knox, 2009). Moreover, the provision of social architecture through camps, extra-curricular activities, meetings of students who share similar goals and facilitated activities during lunch and recess break can be used to build relationships between students.

The extent to which young people feel supported by their teachers was found to be protective of only emotional wellbeing, not mental health. These results are somewhat consistent with US national student surveys, which found students’ connectedness to teachers predicted positive social and emotional health outcomes such as better peer relationships, academic success and reduced participation in health risk behaviours (Resnick, Harris, & Blum, 1993). Teachers can increase their connectedness with their students in the classroom by providing positive feedback and encouragement, active listening, believing in their abilities, caring about them, and providing interactive teaching and learning styles. Engaging with students on a personal level can be achieved through house days, tutor/home room groups and encouraging teachers to get to know first year secondary students as a priority during their duty time.

There are several strengths of this study. Most importantly, the 2-year (four time-points) longitudinal nature of the research design over the transition from primary to secondary school enabled the determination of school climate predictors and mental and emotional wellbeing outcomes during a developmental period that can be challenging for most students. Moreover, these findings are robust due to the low sample attrition rate with 90 % of students completing questionnaires in at least three of the four data collection points. Despite these strengths, there are several limitations to this study. First, the use of self-report of school climate and mental and emotional wellbeing measures could result in some of the associations being due to shared method variance. Peer, teacher or parent reports would be useful in examining these relationships further as the Strength and Difficulties Questionnaires completed by parents and teachers are generally better predictors than those self-completed by adolescents (Goodman, Ford, Simmons, Gatward, & Meltzer, 2000). Safety at school was also measured using a single item. In addition, the baseline data collection (completed at home by primary students prior to their arrival to their secondary schools) was inconsistent with classroom-based data collection procedures used in first and second year of secondary school. To reduce the impact of these differences an explicit and standard protocol (as used in the classroom) was provided to parents for all primary assessments, however parents still may have indirectly or directly influenced their children’s responses to the questionnaire. School climate was measured through factors of school climate rather than directly through an inventory as school climate was not the primary outcome of the original study. The most valid and reliable broad-based measures of whole school climate which can be used by schools to enact practical change (Gangi, 2010) are the Tennessee School Climate Inventory-Revised (SCI-R) (Butler & Alberg, 1991), the Comprehensive School Climate Inventory (CSCI) (Chang, Sandy, & Cohen, 2005), and the Western Alliance for the Study of School Climates School Climate Assessment Instrument (WASSC-SCAI) (Shindler, Taylor, Cardenas, & Jones, 2003). Three comparison secondary schools had a primary school on campus which meant some of their students did not officially change schools during the transition period which may spuriously inflate school climate measures. However, these three comparison secondary schools also had 57 feeder primary schools between them which may limit this effect. Finally, the results may not generalise to other similar aged student populations, as the sample included only Catholic primary and secondary schools within the Perth metropolitan area.

## Conclusion

Few studies have examined longitudinally the relationships between mental and emotional health and the influence of individual-level school climate as students move from primary to secondary school. School climate is not only important for promoting mental and emotional wellbeing among adolescents but also positive behavioural change. School climate factors of feeling safe at school, feeling connected to school, and peer support are all protective of mental and emotional wellbeing over the transition period while connectedness to teachers is protective of emotional wellbeing. Primary school appears to be an important time to establish quality connections to peers who have a powerful role in providing support for one another before the transition to secondary school. However, school policies and practices promoting safety and encouraging and enabling connectedness are important during the first years of secondary school. Regular review and assessment of school policies and practice is recommended to improve school climate and student outcomes.
